# Supplementary material for: Impact of Maternal Fish Consumption on Serum Docosahexaenoic Acid (DHA) Levels in Breastfed Infants: A Cross-Sectional Study of a Randomized Clinical Trial in Japan
Source: Nutrients. 2023 Oct 11;15(20):4338. doi: 10.3390/nu15204338 (PMC10609621; doi:10.3390/nu15204338)
Supplement: Supplementary file 1 [file nutrients-15-04338-s001.zip › nutrients-2536042-supplementary.pdf]

Supplementary Materials

Table S1. Fatty acid composition of cow's milk formula (CMF) (Hohoemi®)

Fatty acid		Weight %
Butyric acid	4:0	0.2
Caproic acid	6:0	0.1
Caprylic acid	8:0	0.3
Capric acid	10:0	0.4
Lauric acid	12:0	4.8
Myristic acid	14:0	3.5
Palmitic acid	16:0	20.7
Stearic acid	18:0	9.2
Palmitoleic acid	16:1n-7	1.8
Oleic acid	18:1n-9	37.4
Linoleic acid	18:2n-6	15.9
α-linolenic acid	18:3n-3	1.6
Arachidic acid	20:4n-6	0.27
Docosahexaenoic acid	22:6n-3	0.40

Some fatty acids were not identified.

Table S2. Fatty acid composition of the elemental formula (EF) (Meiji Elemental formula®)

Fatty acids		Weight %
Palmitic acid	16:0	6.6
Palmitoleic acid	16:1n-7	0.1
Stearic acid	18:0	2.5
Oleic acid	18:1n-9	15.9
Linoleic acid	18:2n-6	60.2
α-linolenic acid	18:3n-3	12.4
Arachidic acid	20:0	0.4
Eicosenoic acid	20:1n-9	0.2
Behenic acid	22:0	0.2
Nervonic acid	24:1n-9	0.1

Some fatty acids were not identified.

Table S3. Nutritional composition of cow's milk formula (CMF) (Hohoemi®) and elemental formula (EF) (Meiji Elemental formula®)

		**CMF (Hohoemi®) Per 100 mL of 13.5% adjusted solution**	**EF (Meiji Elemental formula®) per 100 mL of 17% adjusted solution**
Energy	kcal	68	66
Protein	g	1.5	2
Lipids	g	3.52	0.43
Carbohydrates	g	7.79	13
Sodium chloride amount	g	0.049	0.079
Niacin	mg	0.41	1.0
Pantothenic acid	mg	0.58	0.71
Biotin	µg	0.35	1.6
Vitamin A	µg	53	53
Vitamin B_1	mg	0.054	0.10
Vitamin B_2	mg	0.081	0.15
Vitamin B_6	mg	0.041	0.051
Vitamin B_{12}	µg	0.27	0.68
Vitamin C	mg	9.5	8.5
Vitamin D	µg	0.88	0.90
Vitamin E	mg	0.84	1.0
Vitamin K	µg	3.4	4.3
Folic acid	µg	14	34
Zinc	mg	0.41	0.48
Potassium	mg	66	77
Calcium	mg	51	65
Selenium	µg	1.4	1.8
Iron	mg	0.81	1.1
Copper	mg	43	54
Magnesium	mg	5.4	7.1
Phosphorus	mg	28	37

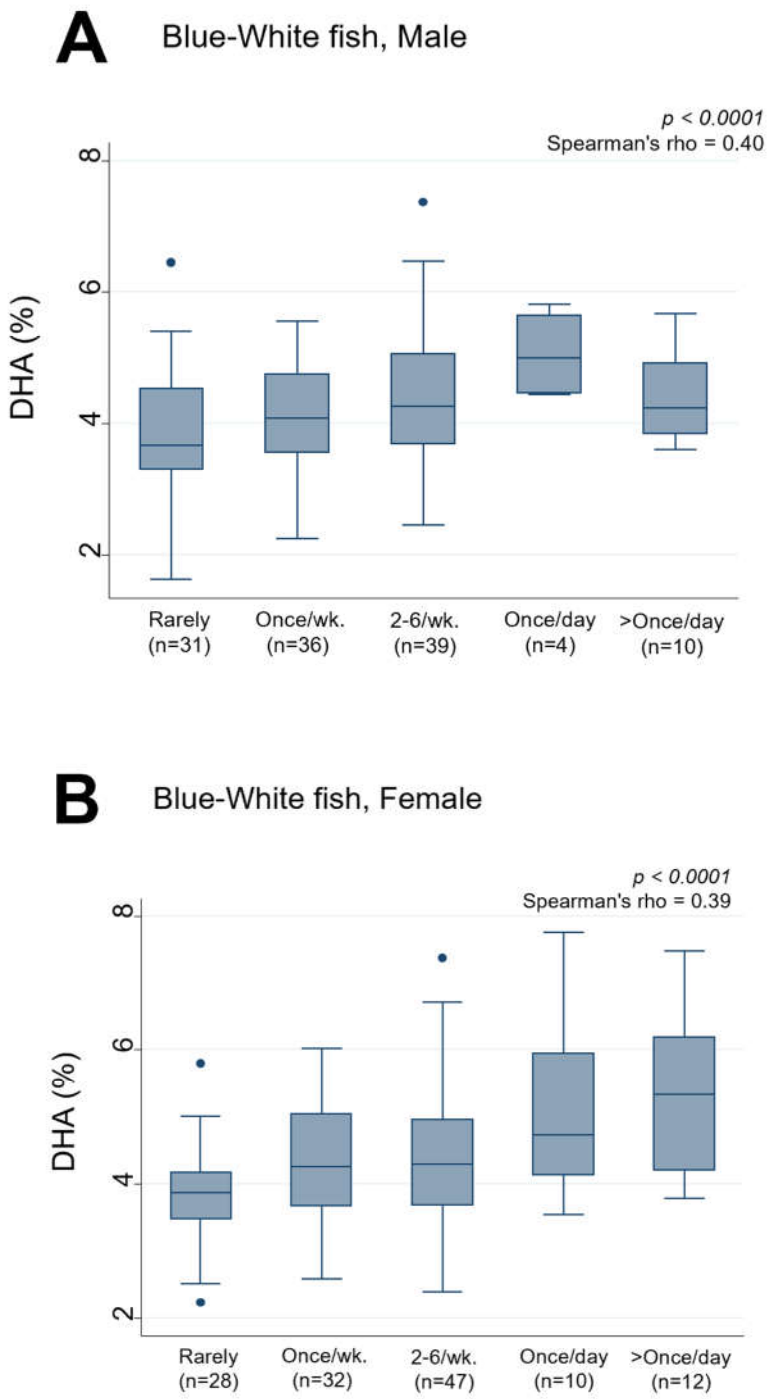

Figure S1. Frequency of Blue-White fish consumption by lactating mothers and the levels of serum DHA (%) in their infants. (A) Males, (B) Females.
